# Addition of fornix transection to frontal-temporal disconnection increases the impairment in object-in-place memory in macaque monkeys

**DOI:** 10.1111/j.1460-9568.2008.06140.x

**Published:** 2008-04-01

**Authors:** C R E Wilson, M G Baxter, A Easton, D Gaffan

**Affiliations:** 1 Department of Experimental Psychology, University of Oxford Oxford, UK; 2 Department of Psychology, University of Durham Durham, UK

**Keywords:** fornix, frontal, hippocampus, inferotemporal, macaque, memory, prefrontal

## Abstract

Both frontal-inferotemporal disconnection and fornix transection (Fx) in the monkey impair object-in-place scene learning, a model of human episodic memory. If the contribution of the fornix to scene learning is via interaction with or modulation of frontal-temporal interaction − that is, if they form a unitary system − then Fx should have no further effect when added to frontal-temporal disconnection. However, if the contribution of the fornix is to some extent distinct, then fornix lesions may produce an additional deficit in scene learning beyond that caused by frontal-temporal disconnection. To distinguish between these possibilities, we trained three male rhesus monkeys on the object-in-place scene-learning task. We tested their learning on the task following frontal-temporal disconnection, achieved by crossed unilateral aspiration of the frontal cortex in one hemisphere and the inferotemporal cortex in the other, and again following the addition of Fx. The monkeys were significantly impaired in scene learning following frontal-temporal disconnection, and furthermore showed a significant increase in this impairment following the addition of Fx, from 32.8% error to 40.5% error (chance = 50%). The increased impairment following the addition of Fx provides evidence that the fornix and frontal-inferotemporal interaction make distinct contributions to episodic memory.

## Introduction

The hippocampus and surrounding regions of cortex have been proposed as a unitary medial temporal lobe memory system for declarative memory (Squire & Zola-Morgan, 1991). Recent experiments in both experimental animals and in human patients with brain lesions have questioned this view, and proposed that declarative memory arises from a much wider network within the brain. First, lesions that do not affect the integrity of medial temporal lobe structures can nevertheless result in severe amnesia, for example disconnection of frontal and inferotemporal cortex (Browning *et al*., 2005, 2007; Wilson *et al*., 2007), disconnection of the inferotemporal cortex from subcortical structures (Easton & Gaffan, 2001; Easton *et al*., 2002), or interruption of white matter connections of the temporal lobes (Gaffan *et al*., 2001). Second, lesions limited to individual structures within the medial temporal lobe, for example the perirhinal cortex or hippocampus, produce cognitive impairments that are perceptual as well as mnemonic in nature (Buckley *et al*., 2001; Bussey *et al*., 2002, 2003; Barense *et al*., 2005; Lee *et al*., 2005). Taken together, these data support a different view of the organization of memory in the brain, that it arises from a network of cortical and subcortical interactions, and that perceptual and mnemonic functions occur together within brain structures (Horel, 1978; Gaffan, 2002, 2005; Murray *et al*., 2007).

In monkeys, the object-in-place scene-discrimination task (Gaffan, 1994b) has been used to test an analogue of human episodic memory. Like memory for events in humans, this task requires memory for both object and spatial elements, and the scenes are acquired very rapidly, often after a single presentation. The object-in-place task is impaired in monkeys with transection of the fornix, a major input–output pathway of the hippocampus (Gaffan, 1994b). The task is also impaired by disconnection of the frontal cortex from the inferotemporal cortex (Browning *et al*., 2005) or indeed a partial version of the same disconnection (Wilson *et al*., 2007). These results raise the question whether the fornix subserves and modulates the interaction of the frontal cortex with the inferotemporal cortex, or whether it performs a completely separate role. In order to distinguish between these two possibilities we trained three rhesus monkeys on the object-in-place scene-learning task, tested their performance following frontal-inferotemporal disconnection, and then retested them following the addition of fornix transection (Fx) to that disconnection. If the fornix supports frontal-inferotemporal interaction in scene learning as part of a unitary system, then the addition of Fx should not increase the impairment noted following frontal-inferotemporal disconnection. Such logic has previously been used to demonstrate the functional unity fornix-mammillary system (Parker & Gaffan, 1997b). In contrast, an increase in impairment following the addition of Fx would signal that the two have independent roles in episodic memory.

## Materials and methods

### Subjects

Three male rhesus monkeys (*Macaca mulatta*, M1–M3), mean age 3.4 years and mean weight 4.4 kg at the beginning of behavioural training, were subjects in this study. Monkey M3 was housed socially in a troop, in indoor enclosures attached to standard caging; monkeys M1 and M2 were pair housed in standard caging at the time of testing. Monkeys M1 and M2 were described as monkeys A and B, respectively, in Browning *et al*. (2005), and so their data from disconnection but not Fx has been previously reported. Monkey M3 has not been reported previously. Intended lesions in all three cases were identical, and for the sake of clarity are described below.

Water was available *ad libitum* in the home enclosure; each monkey's daily food ration was delivered in the test box, and was supplemented with fruit and forage mix in the home enclosure. All experimental procedures were conducted under the authority of personal and project licences held by the investigators in compliance with the UK Animals (Scientific Procedures) Act 1986.

### Apparatus

Each monkey was wheeled from the home enclosure into the test cubicle in a transport cage, which was fixed in front of a video-display unit with a touch-sensitive screen (380 × 280 mm, 800 × 600 pixel resolution). The monkey could reach through horizontal bars at the front of the cage to touch the screen and retrieve rewards. Stimulus presentation, recording of touches to the screen and reward delivery were all under computer control. A pellet dispenser delivered 190 mg banana-flavoured or sugar pellets (P. J. Noyes, Lancaster, NH, USA) into a hopper located below the touch screen. A metal ‘lunchbox’ (approximately 200 × 100 × 100 mm) was located to the left of the hopper and was filled with the ‘large food reward’, which consisted of a mixture of wet monkey chow, banana, apple, orange, nuts, seeds and dates. Cameras positioned at different locations within the test cubicle permitted observation of the monkey while he was performing the task. The entire apparatus was located in an experimental cubicle that was dark except for the illumination of the video screen.

### Behavioural testing

The object-in-place scene-learning task was adapted from Gaffan (1994b), and was identical to that reported in previous studies (Browning *et al*., 2005; Wilson *et al*., 2007). Each trial consisted of an artificially constructed scene that occupied the whole area of the display screen. Two foreground ‘objects’, comprising small, randomly selected and coloured typographic characters, were each placed in a constant location in the scene. Behind these objects, the backgrounds were generated using an algorithm that drew a random number (between two and seven) of randomly located ellipses and ellipse segments of random colour, size and orientation on a randomly coloured initial background, and then drew a single very large randomly selected typographic character, clearly distinct in size from the foreground objects, in a random colour somewhere in the scene. All of the colours were assigned with the constraint that the foreground objects should be visible, and so to achieve this there was a minimum separation in colour space between the colours of a foreground object and the colour of any element of its local background. In each scene, one of the two foreground objects was the correct one for the monkey to touch and the other was incorrect. Because these scenes were generated by an algorithm based on a random number generator, an infinite number of unique scenes could be generated. For example stimuli, see Browning *et al*. (2005) and Gaffan (1994b).

In an initial shaping procedure, each monkey learned to touch single foreground objects against a black background. Once they had mastered this, the same task was continued but now with additional scene elements added gradually over a number of sessions, until the monkey reliably touched the single foreground object when presented with a new scene. We then introduced learning problems with two foreground objects (one correct and one incorrect, as described above). The monkeys also now received a number of repetitions of each scene within a session, in order to allow them to learn about the set of scenes. Monkeys never received the same scene again on a subsequent day, and therefore all scene learning was within a session. The number of repetitions and number of scenes was controlled by the experimenter on the basis of the monkey's progress, and gradually increased to the final level. In the final version of the task, 20 new scenes were presented in each session, and the list of 20 scenes was repeated eight times. A touch to the correct object caused the object to flash for 2 s, then the screen blanked and a reward pellet was delivered. A touch to the incorrect object caused the screen to blank immediately. For the first repetition of the list of scenes only, incorrect responses were followed by a correction trial in which the scene was re-presented with only the correct object present. Touches anywhere else in the scene caused the screen to blank and the trial was repeated. Touches to the screen during the inter trial interval re-set that interval. When the monkey completed the final trial of a session the lunchbox opened automatically, and the monkey received the large food reward. If the final trial was incorrect, a correction trial was given so that the monkey only ever received the large food reward following a correct response.

Once performance on the scenes task stabilized, each monkey was given a 2-week period of rest, after which he was given 12 daily sessions of testing. Data from the final 10 sessions of this test constituted the preoperative performance test. In the experiment, the monkeys received four such performance tests, at the four stages of the experiment, which were once prior to any surgery, and once following a minimum 2-week recovery period after each of the three surgeries. The performance test at the second of these stages, following a unilateral frontal lobe ablation, was designed as a test for any general effects of surgery or a break in testing. Henceforth the four stages of the experiment, at each of which the monkeys completed a performance test, will be referred to, in order of completion, as Pre-Op, Unilateral FL (frontal lobe ablation), FLxIT (frontal-inferotemporal disconnection) and FLxIT + Fx (frontal-inferotemporal disconnection with fornix transection).

### Surgery

**Fig. 1:**
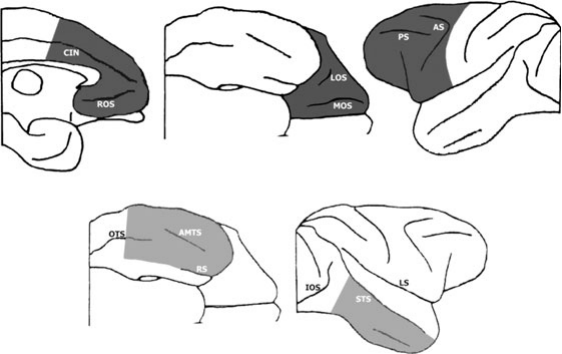
Representation of the intended extent of the ablation of frontal cortex (in the left hemisphere, top row, dark grey) and the inferotemporal cortex (in the right hemisphere, bottom row, light grey) shown from ventral, lateral and, in the frontal case, medial views. The shaded areas indicate the areas of intended removal. AMTS, anterior middle temporal sulcus; AS, arcuate sulcus; CIN, cingulate sulcus; IOS, intraoccipital sulcus; LOS, lateral orbital sulcus; LS, lateral sulcus; MOS, medial orbital sulcus; OTS, occipitotemporal sulcus; PS, principal sulcus; ROS, rostral sulcus; RS, rhinal sulcus; STS, superior temporal sulcus.

Neurosurgical procedures were performed in a dedicated operating theatre under aseptic conditions. Each monkey's first neurosurgical procedure consisted of a left unilateral frontal lobe ablation, their second procedure was a right unilateral inferotemporal cortex ablation, and their third procedure was a bilateral transection of the fornix. Previous studies have demonstrated that there is no effect of the side on which the unilateral ablations are performed, in this task and others (e.g. Gaffan *et al*., 2002; Browning *et al*., 2005, 2007). The intended extents of the frontal and inferotemporal ablations are shown in Fig. 1. Surgical procedures for frontal-temporal disconnection in cases M1 and M2 are described in the earlier report (Browning *et al*., 2005). Case M3 has not been previously reported. All surgical procedures (frontal-temporal disconnection and Fx in case M3, and Fx in cases M1–M3) took place under general anaesthesia (sodium thiopentone, i.v., to effect in cases M1 and M2, and isoflurane, 1–2.75%, to effect, in 100% oxygen in case M3).

Monkeys were maintained in a state of deep anaesthesia during surgery by monitoring pulse rate, blood oxygenation, body temperature and peripheral reflexes, consistent with United Kingdom Home Office regulations. Perioperative care included administration of antibiotics and analgesic drugs, consistent with veterinary advice and Home Office guidelines. The frontal-inferotemporal disconnection in case M3 was performed exactly as described in Browning *et al*. (2005). The first stage of this surgery was intended to remove the whole of the frontal cortex in the left hemisphere, except for the primary motor cortex, leaving the underlying white matter and corpus striatum intact. The second stage (after recovery and postoperative testing following the first surgery) removed the inferotemporal cortex in the right hemisphere, extending from the fundus of the superior temporal sulcus to the fundus of the rhinal sulcus. The posterior part of the lesion included both banks of the anterior part of the occipitotemporal sulcus. The posterior limit of the lesion was a line perpendicular to the superior temporal sulcus, 5 mm anterior to the inferior occipital sulcus. The anterior limit of the lesion was bounded by a line drawn from the anterior tip of the superior temporal sulcus around the temporal pole to the tip of the rhinal sulcus. We removed all cortex within these limits. This included cortex within both the anterior and posterior middle temporal sulci.

For the Fx, the dura mater was cut to expose the hemisphere up to the midline, and veins draining into the sagittal sinus that impeded access to the midline were cauterized and cut. The right hemisphere was retracted from the falx with a brain spoon. A glass aspirator was used to make a sagittal incision no more than 5 mm in length in the corpus callosum at the level of the interventricular foramen. The fornix was sectioned transversely by electrocautery and aspiration with a 20-gauge metal aspirator, which was insulated to the tip.

### Histology

After completion of behavioural training each monkey was sedated with ketamine (10 mg/kg), deeply anaesthetized with intravenous barbiturate and transcardially perfused with 0.9% saline followed by 10% formalin. The brain was cryoprotected in formalin-sucrose and then sectioned coronally on a freezing microtome at 50 µm thickness. A 1-in-10 series of sections through the area of the lesions was mounted on gelatin-coated glass microscope slides and stained with Cresyl violet.

**Fig. 2:**
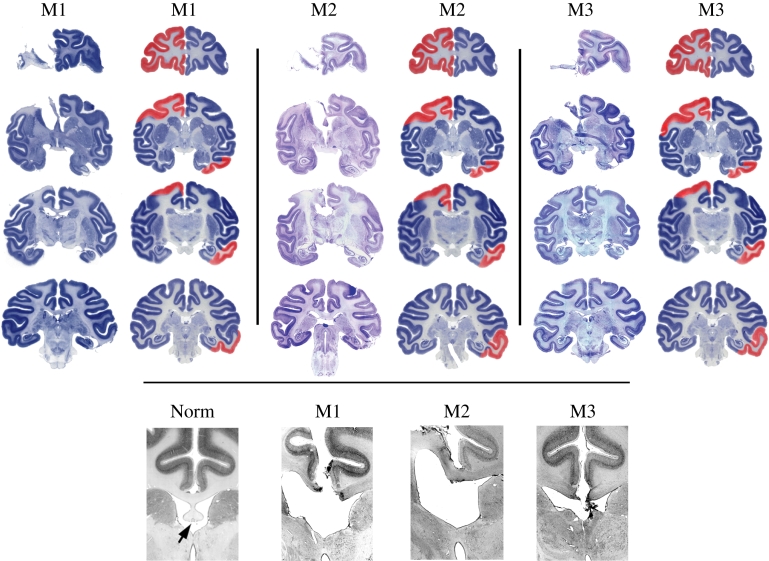
Histological sections displaying the lesions in all three monkeys. Top half: coronal sections of the actual and reconstructed lesions of the three monkeys. The first column shows four actual Cresyl violet-stained sections at different levels taken from monkey M1. The second column shows a reconstruction coloured in red of the extent of this ablation represented on Cresyl violet-stained sections taken from a normal macaque brain. The remaining columns show similar sections and reconstructions for monkeys M2 and M3. Bottom half: detail from a coronal section from the brain of a normal monkey (Norm) where the arrow indicates the intact fornix, and similar sections from monkeys M1–M3 where the fornix has been completely transected at the level of the interventricular foramen.

Figure 2 illustrates the lesions in the three monkeys. The upper section of the figure shows four actual sections and their corresponding reconstructions on a normal monkey brain from monkey M1, along with reconstructions from monkeys M2 and M3 at the same anterior–posterior level. This method of displaying the histology better illustrates the size of the lesions, in particular the removed sulci that may not be obvious from the original sections due to collapse of overlying cortex. This upper section of Fig. 2 shows the unilateral frontal and inferotemporal ablations. These reconstructions show that, whilst there was slight sparing of posterior medial prefrontal cortex (PFC) in monkeys M1 and M3, the overall extent of the frontal ablations was as intended. With the exception of partial sparing of the lower bank of the superior temporal sulcus in monkey M1, the lesions to the inferotemporal cortex were also complete. Hence the ablations at stage FLxIT were as intended and shown in Fig. 1.

**Fig. 4:**
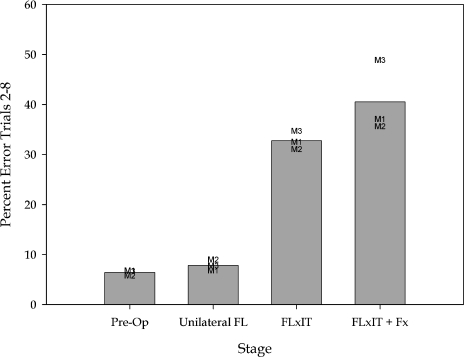
Average percent error in Trials 2–8 over the four stages. The bars represent the mean percent errors committed by the three monkeys on Trials 2–8 in the 10 critical sessions of the performance test at that particular stage. The scores of the individual monkeys (M1–M3) that contributed to that mean are also represented. All of these data correspond to those in Table 1.

The bottom part of Fig. 2 shows detail from coronal sections of a normal monkey (EX), indicating the location of the intact fornix, and monkeys M1–M3. Microscopic examination of the stained sections revealed in every case a complete section of the fornix with no damage outside the fornix, except for the incision in the corpus callosum of each animal as described in the surgical procedures, and damage to the cingulate gyrus resulting from the unilateral frontal ablation. The callosal incision of monkey M3 was of no greater extent than that of the other two monkeys, and there was no incidental damage around the corpus callosum in this animal. Therefore the damage caused by Fx in M3 was in no way more extensive than in the other two monkeys, and so incidental damage cannot account for the difference in magnitude of impairment between M3 and the other two monkeys that can be seen in Fig. 4.

**Table 1:** Data from the four stages of the experiment for each of the three monkeys

	Mean percentage error, Trials 2–8			
	
	Stage 1	Stage 2	Stage 3	Stage 4
				
Monkey	Pre-Op	Unilateral FL	FLxIT	FLxIT + Fx
M1	6.79	6.79	32.50	37.07
M2	5.79	9.00	31.07	35.64
M3	6.71	7.79	34.71	48.86
Mean	6.43	7.86	32.76	40.52

The data presented are mean percent error for each monkey on Trials 2–8 over the 10 critical days of the performance test carried out at each of the four stages. These data are presented graphically in Fig. 4.

## Results

**Fig. 3:**
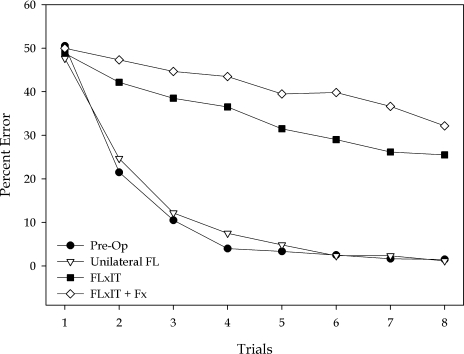
Learning curves for within-session learning of object-in-place problems for the four stages. The mean scores for the three monkeys are represented at stages Pre-Op (filled circles), Unilateral FL (open triangles), FLxIT (filled squares) and FLxIT + Fx (open diamonds). The graph shows the rapid speed with which monkeys acquired these problems preoperatively, and the impaired learning after the disconnection, and further after the addition of Fx.

The learning rate in object-in-place learning was measured at each stage as the mean number of errors made in Trials 2–8 in the final 10 sessions of each performance test. Table 1 shows the learning rates for these sessions expressed as percent error for all three monkeys at each of the four stages in the present experiment. The learning curves for the monkeys at the four stages are displayed graphically in Fig. 3, whilst the mean percent error at each stage is shown in Fig. 4. In both cases they demonstrate the small change between stages Pre-Op and Unilateral FL, but a large increase in percent error at stage FLxIT, and a further increase in percent error in each monkey at stage FLxIT + Fx.

We tested the overall effect of the experimental stage on the mean percent error in Trials 2–8 of the 10 critical sessions of each performance test. A repeated-measures anova, with experimental stage as a factor, revealed a significant effect, *F*_3,6_ = 73.661, *P* < 0.0005. The Unilateral FL stage of the experiment provided a within-subjects control for the effects of a surgical procedure and a break in testing. We performed a designed comparison using the pooled error term to look for differences in performance between stages Pre-Op and Unilateral FL, and found no such difference, *t*_6_ = 0.501, *P* = 0.317 one tailed, confirming no significant effect of either the break in testing or the unilateral lesion on the task. We performed a further designed comparison between stages Unilateral FL and FLxIT, showing a significant effect of the completion of the disconnection, *t*_6_ = 8.719, *P* < 0.0005 one tailed. This is the result of Browning *et al*. (2005). Finally, in the critical test for the current experiment, we performed a final designed comparison between stages FLxIT and FLxIT + Fx, revealing a significant effect, *t*_6_ = 2.718, *P* = 0.0174 one tailed. Thus, we have shown a significant effect of the addition of Fx to frontal-inferotemporal disconnection.

The effect of addition of Fx to frontal-temporal disconnection is very clearly significant, but the difference is small when compared with the change following disconnection. Given the within-subjects design of the current study, one interpretation of this effect might be that the additional surgical procedure alone was sufficient to cause the increased impairment. We regard this as highly unlikely. First, surgery is insufficient to cause impairment in the current task between stages Pre-Op and Unilateral FL, so it is unclear why it would cause impairment at stage FLxIT + Fx. Second, we have previously shown that monkeys with repeated surgeries can show no behavioural impairment on the current task. For example, the control subject S4 reported by Wilson *et al*. (2007) received two surgeries and yet was still performing better after those two operations (12.43% error) than preoperatively (15.43% error). Furthermore, this same monkey received a further surgery (unpublished observations) and after three surgical operations, the same number as the monkeys in the current experiment, this monkey was still performing the task at the same level as preoperatively (15.21% error).

**Fig. 5:**
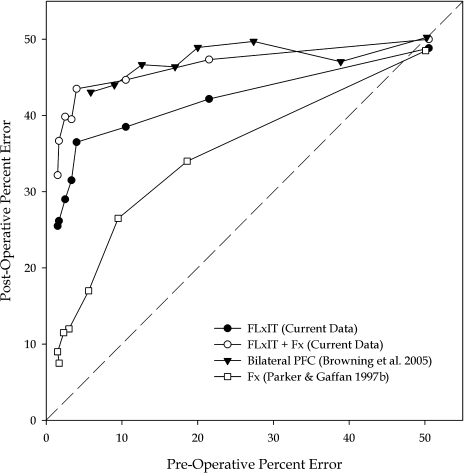
Phase-space plot comparing the current data with those presented in Browning *et al*. (2005) and Parker & Gaffan (1997b). Data are plotted as mean preoperative percent error against mean postoperative percent error, which allows a comparison of the results of the two studies across a range of preoperative performance, accounting for any possible variation in preoperative performance between the studies. Each point represents the mean pre- vs postoperative performance on a given repetition of lists of new scenes. Therefore, in all four conditions the point representing the first experience of a new set of scenes shows chance performance of approximately 50% in both pre- and postoperative phases, as this is the first time the monkeys see the set of scenes. The dashed diagonal line represents performance that is identical prior to and after surgery. Points below this line represent an improvement from pre- to postoperative performance tests, and points above it represent an impairment in performance from pre- to post-. As learning progresses through the eight repetitions of the scenes, the graph displays the extent to which the improvement with each successive repetition is similar or different pre- and postoperatively. Data for FLxIT are those following the second surgeries in the disconnection procedure, and include two monkeys presented in group FLxIT by Browning *et al*. (2005). The FLxIT + Fx group represents data following the monkeys' third surgery in the current experiment, and the Bilateral prefrontal cortex (PFC) following their only surgery in Browning *et al*. (2005). Data from group Fx are those following a second surgery in those monkeys, the first surgery being a sham lesion that had no behavioural effect on this task (see right panel, fig. 1, Parker & Gaffan, 1997b). The FLxIT group shows worse performance than that of group Fx, but both show a postoperative impairment. The FLxIT + Fx group shows similar performance to group Bilateral PFC.

The comparison of the current results with those from the same task reported previously is informative, and because the testing procedure for the monkeys with FLxIT + Fx is identical to monkeys with bilateral PFC lesions tested by Browning *et al*. (2005), and monkeys with Fx alone tested by Parker & Gaffan (1997b), a direct comparison of their performance can be made. Figure 5 shows a phase-space plot, in which the postoperative and preoperative learning rates are plotted against each other. The strength of this plot is that it displays the postoperative results of the three studies across a range of preoperative performance levels. This means that a comparison can be made between the studies without concern over any differing levels of preoperative performance between them. Figure 5 shows the effect alone of FLxIT in the current study, in which two animals were the same as those reported in group FLxIT by Browning *et al*. (2005), and also the effect alone of bilateral Fx in Parker & Gaffan (1997b), in which the monkeys had received a control lesion without behavioural effect on the task prior to Fx. Both of these lesions impair the task, albeit to differing degrees. Figure 5 also shows the increased postoperative impairment following FLxIT + Fx, and demonstrates that at this stage the monkeys' performance was very similar to that of monkeys with bilateral ablation of PFC in Browning *et al*. (2005). Indeed a one-way anova comparing the performance of the current monkeys at stage FLxIT + Fx and the Group PFC in table 1 of Browning *et al*. (2005) revealed no significant difference between the two groups, *F*_1,5_ = 2.745, *P* = 0.158.

## Discussion

We have shown that the addition of Fx to monkeys who had received a disconnection of frontal and inferotemporal cortex significantly increases impairment on the object-in-place task. Following FLxIT + Fx, the severe impairment in object-in-place scene learning is not significantly different from that of a group of monkeys with a bilateral PFC ablation.

The additive effect of Fx with FLxIT disconnection argues that fornix and frontal-inferotemporal interaction make contributions to episodic memory that are, at least to some extent, distinct. There is no guarantee that two lesions that each individually impair task performance will produce an additive effect when they occur together. For example, lesions of the fornix and mammillary bodies each produce a moderate impairment in scene learning. However, the effect of these two lesions together is identical to the effect of each of the two lesions alone (Parker & Gaffan, 1997b). This implies that the fornix and mammillary bodies form part of the same functional system, as lesions to one of these structures leaves the other without function. This was not the case in the present experiment: the addition of a fornix lesion, which produces a moderate impairment in scene learning on its own, to frontal-temporal disconnection, produced an additional decrement in performance. Consequently, it can be concluded that the functions of the fornix and of frontal-temporal interaction in scene learning are, to some degree at least, distinct from one another.

One implication of this finding is that the fornix cannot be the sole route for interaction between inferotemporal and frontal cortex. The fornix is the major input–output pathway of the hippocampus, and forms a unitary system with the mammillary bodies as part of a proposed cortico-cortical association pathway for episodic memory (Parker & Gaffan, 1997b). These authors hypothesized that the output of this system reached frontal cortical structures via projections to the anterior thalamus (Goldman-Rakic & Porrino, 1985; Amaral, 1987), creating a cortico-cortical network for episodic memory. Effects of anterior thalamic lesions on scene learning are consistent with this hypothesis (Parker & Gaffan, 1997a). Nevertheless, this hypothesis would predict that the addition of Fx to a direct disconnection of frontal and inferotemporal cortex would produce no additional impairment in behaviour, which is contrary to the result of the current investigation. Therefore, connections through the fornix must be making a contribution to scene learning distinct from supporting intrahemispheric interactions of frontal and inferotemporal cortex. That it can operate in part independently in this way, of course, supports the idea that the networks supporting episodic memory in the brain are both diverse and widespread.

One possibility, then, is that frontal-temporal interaction and fornix provide two completely independent pathways subserving different aspects of scene learning. We do not wish to argue that this is the case, or to argue that, in the intact brain, the fornix does not normally subserve some of the learning that is lost in a disconnection between frontal and inferotemporal cortex. Indeed, we know that there is some interaction between the areas disconnected by frontal-inferotemporal disconnection and the fornix, because disconnection of perirhinal cortex (which is included in the current inferotemporal lesions) and fornix causes a significant impairment in object-in-place scene learning (Gaffan & Parker, 1996). This impairment is of a similar magnitude to that following bilateral lesions of the fornix, mammillary bodies and anterior thalamic nuclei in the studies cited above. As Gaffan & Parker (1996) argued, the differences in effects on a range of other tasks between bilateral fornix and perirhinal lesions (e.g. Gaffan, 1994a) show that in some tasks these two function independently. But in the case of object-in-place scene learning, their interaction is of functional importance. This implies that the independent effect that we have inferred from the current data is not the only role of the fornix in the current task, and will therefore not provide a complete explanation for the impairment in the task following bilateral Fx. Nevertheless, we need to describe the particular element of scene learning carried out by the fornix that is independent of frontal-inferotemporal interaction.

One possibility is that section of the fornix eliminates a pathway by which interhemispheric frontal-inferotemporal interactions take place, that can support scene learning after crossed unilateral lesions of frontal and inferotemporal cortex eliminate intrahemispheric interaction. This possibility can probably be dismissed because Fx spares commissural connections of the hippocampus (Demeter *et al*., 1985) and additional pathways for interhemispheric frontal-inferotemporal interaction exist, for example via frontothalamic connections (Preuss & Goldman-Rakic, 1987).

A more likely hypothesis is that connections between the subiculum and PFC, carried by the fornix (Saunders & Aggleton, 2007), support aspects of scene learning that are independent of frontal-inferotemporal interaction. We may theorize that information from the dorsal visual stream reaches the hippocampus via parahippocampal cortical areas (Suzuki & Amaral, 1994) that are not included in the inferotemporal ablation, providing a route for visual information to reach the PFC despite the presence of crossed unilateral lesions of frontal and inferotemporal cortex. Obviously this pathway is not sufficient to support normal scene learning in the presence of frontal-inferotemporal disconnection, but the present data suggest that it may make a contribution to scene learning nevertheless. That the critical pathway for scene learning that moves through the fornix does involve the PFC is suggested by the observation that the magnitude of impairment after FLxIT + Fx is identical to the impairment caused by bilateral prefrontal ablation.

What are the distinct contributions of frontal-inferotemporal interaction and fornix to scene learning? Frontal-inferotemporal interaction in visual learning has been suggested to be necessary for the representation of temporally complex events (Browning *et al*., 2005, 2007). For example, in scene learning, the requirement to integrate visual information across saccades, in order to form a representation of the entire visual scene to support discrimination learning, creates a temporally complex event that must be represented by the PFC. Thus, damage to the PFC, or disconnection of the frontal cortex from the inferotemporal cortex (where complex visual information is represented), produces a devastating impairment in scene learning. Importantly, this impairment cannot be ascribed uniquely to perseveration (Browning *et al*., 2005). In a very different task, discrimination learning set, frontal-inferotemporal disconnection also produces a devastating impairment (Browning *et al*., 2007), because performance in this task requires the formation of prospective memories, which are also representations of temporally complex events. This view is supported by the observation that visual discrimination learning that does not require the use of prospective memories, or the integration of information from different views of a visual scene, is unaffected by frontal-inferotemporal disconnection (Parker & Gaffan, 1998; Gaffan *et al*., 2002). The effect of frontal-inferotemporal disconnection in this task is congruent with the idea that detailed visual representations that occur in inferotemporal cortex are critical for the representation of temporally extended events in the PFC that are required for efficient scene learning.

The observation that the addition of Fx to frontal-inferotemporal disconnection produces an additional impairment in scene learning − one that reaches the level of impairment caused by bilateral prefrontal lesions − suggests that subcortical afferents to cortex in the fornix could subserve interaction between PFC and visual information from outside the inferotemporal cortex, perhaps, as suggested, via dorsal stream projections to the parahippocampal cortex. Further, it suggests that this information may also be involved in representation of temporally extended events within the PFC that contribute to efficient scene learning. Critically, the present results suggest that both sources of visual information are important for scene learning: that is, both detailed visual object representations within the inferotemporal cortex (Tanaka, 1996) as well as spatial aspects of vision that are represented in the dorsal visual stream, which include information about object shape as well as localization (Sereno & Maunsell, 1998; Lehky & Sereno, 2007). Neither source of visual information can support scene learning on its own, because significant impairments in scene learning arise after both Fx and frontal-inferotemporal disconnection individually. Further, neither pathway is completely independent of the other, as a disconnection of parts of the inferotemporal cortex from the fornix causes an impairment on the task (Gaffan & Parker, 1996).

Thus, scene learning − and, by extension, episodic memory − requires the integration of information about the spatial organization of visual objects as well as details about their visual properties, characteristics that are associated with the dorsal and ventral visual streams, respectively. This speculative hypothesis suggests a number of experimental tests of the contributions of the different types of visual information to episodic memory, and these will be the topics of future studies. Whether or not this particular hypothesis is true, the current result reinforces the argument that episodic memory for scenes is not a function restricted to a discrete memory system, but rather is supported by a widespread cortical and subcortical network.

## Abbreviations

FL: frontal lobe
FLxIT: frontal-inferotemporal disconnection
Fx: fornix transection
IT: inferotemporal cortex
PFC: prefrontal cortex.
